# The Lattice Model of Particles with Orientation-Dependent Interactions at Solid Surfaces: Wetting Scenarios

**DOI:** 10.3390/ijms232112802

**Published:** 2022-10-24

**Authors:** Andrzej Patrykiejew

**Affiliations:** Department of Theoretical Chemistry, Institute of Chemical Sciences, Faculty of Chemistry, MCS University, 20031 Lublin, Poland; andrzej.patrykiejew@mail.umcs.pl

**Keywords:** wetting phemomena, lattice model, orientation-dependent interactions, Montr Carlo simulation

## Abstract

Wetting phenomena in a lattice model of particles having two chemically different halves (A and B) and being in contact with solid substrates have been studied with Monte Carlo methods. The energy of the interaction between a pair of neighboring particles has been assumed to depend on the degree to which the AA, AB and BB regions face each other. In this work, we have assumed that $u_{AA}=-1.0$ and considered three series of systems with $u_{AB}=u_{BB}$, $u_{AB}=0$ and $u_{BB}=0$. The phase behavior of bulk systems has been determined. In particular, it has been shown that at sufficiently low temperatures the bulk systems order into the superantiferromagnetic (SAF) phase, or into the antiferromagnetic (AF) phase, depending on the magnitudes of AA, AB and BB interaction energies, $u_{AA}$, $u_{AB}$ and $u_{BB}$. The SAF structure occurs whenever $\epsilon =u_{AA}+u_{BB}-2u_{AB}$ is lower than zero and the AF structure is stable when ϵ is greater than zero. The wetting behavior has been demonstrated to depend strongly on the structure of the bulk condensed phase, the interactions between fluid particles and the strength of the surface potential. In all series, we have found the dewetting transition, resulting from the limited stability of different ordered structures of surface phases. However, in the systems that exhibit the gas–liquid transition in the bulk, the reentrant wetting transition has been observed at sufficiently high temperatures. The mechanism of dewetting and reentrant wetting transitions has been determined. Moreover, we have also demonstrated, how the dewetting transition in the series with $u_{AB}=0$ is affected by the wall selectivity, i.e., when the interaction between the parts A and B of fluid particles and the solid is different.

## 1. Introduction

A lot of effort has been devoted to the multilayer adsorption and wetting phenomena over the last five decades. revealing plenty of diverse phenomena [1,2,3,4,5,6,7,8,9,10,11,12,13,14,15]. In general, there are three main scenarios of molecular film growth on solid substrates [9], usually termed Frank–van der Merwe (type-1), Stranski–Krastanov (type-2) and Volmer–Weber (type-3) growth modes [10]. In the type-1 growth, the films grow asymptotically toward the infinite thickness, when the pressure approaches the bulk coexistence. In the case of type-2 growth, the films reach the finite thickness, usually limited to only a few molecular layers. This situation is referred to as the incomplete or partial wetting. Finally, when the film exhibits type-3 growth mode, the adsorption remains very small at any pressure, up to the bulk coexistence, and it corresponds to nonwetting.

Complete wetting requires the adsorbate–substrate interaction to be sufficiently strong, and when it dominates over the adsorbate–adsorbate interaction, complete wetting may occur at any temperature, even at T=0 [2,3,6,7,9]. However, the adsorbed films may grow in different ways at low and high temperatures. At low temperatures, the film thickness usually increases in a step-wise manner, via a series of first-order layering transitions [6,7,9]. Every layering transition may lead to the condensation of a single molecular layer or it may involve a simultaneous condensation of a certain number of molecular layers, depending on the magnitudes of the adsorbate–adsorbate and the adsorbate–substrate interaction energies and the temperature [9,16]. In any case, each layering transition terminates in the respective critical point, $T_{c}\left(n\right)$ [6,9], with *n* being the layer number and $T_{c}\left(n\right)$ converges to the roughening temperature, $T_{R}$ [17], when *n* goes to infinity. At the temperatures above $T_{R}$, the interface between the adsorbed film and the bulk strongly fluctuates and hence the film thickness increases continuously and ultimately diverges at the bulk coexistence.

When the strength of surface potential is lowered below a certain threshold value, complete wetting may occur only at finite temperatures, starting at the wetting temperature $T_{w}$ [4,6,9]. Below $T_{w}$, the film thickness remains finite at any pressure up to the bulk coexistence and hence such systems show type-2 or type-3 growth.

The actual mode of the film development is controlled by the relation between the wetting and roughening temperatures [6,9]. As long as $T_{w}<T_{R}$, the interface between the film and the bulk phase is sharp and layering transitions may still occur, but only at temperatures between $T_{w}$ and $T_{R}$. On the other hand, when $T_{w}>T_{R}$, the film develops differently. Instead of layering transitions, it usually exhibits the prewetting, or the thin-thick film, transition, before the wetting transition at the bulk coexistence. In such cases, the wetting is the first order transition [3]. A further weakening of the substrate potential has been predicted [3,9,18] to lead to the continuous wetting transition (critical wetting) at the bulk coexistence. Ultimately, when the surface potential becomes weak enough, only incomplete wetting (type-2) or non-wetting (type-3) behavior occurs at any temperature.

The experimental studies of wetting of simple gases on graphite [19] demonstrated that the relative strength of adsorbate–adsorbate and adsorbate–substrate interactions is not the sole factor that determines the wetting behavior. It was demonstrated, however, that at sufficiently low temperatures, the type-1 growth occurs over a certain range of relative strength of the adsorbate–adsorbate and adsorbate–substrate interactions. The type-2 behavior was found to occur for both sufficiently weak and sufficiently strong surface potentials. It was shown [20,21,22] that the reentrant type-2 growth may result from a mismatch between the lattices of the solid-like film and the bulk solid phase. Another frequently observed phenomenon is triple point wetting [23,24], which occurs when the solid phase does not wet the surface while the liquid phase does. There are several possible scenarios of wetting close to the bulk triple point [8], depending on the wetting properties of the solid and liquid bulk phases, as well as the structure of the interface between the surface and bulk phases. On the other hand, it was theoretically predicted by Cahn [25] and by Saam and Ebner [26] that complete wetting should occur at temperatures below the bulk critical temperature. This is so because the surface tension goes to zero at temperatures lower than the bulk critical temperature, and hence complete wetting should occur. Another theoretically predicted and observed experimental phenomenon is reentrant wetting [8,27,28,29,30,31,32,33,34]. It occurs when the adsorbed film and/or the bulk condensed phase change their structure when the temperature changes.

In the recent paper [35], we presented a rather simple lattice gas model of particles with orientation-dependent interactions in contact with a solid substrate. The discussion was limited to the systems with the adsorbate–adsorbate interaction characteristic of Janus-like particles [36], composed of the two halves, A and B. The energy of AA interaction was assumed to be attractive and fixed ($u_{AA}=-1.0$), the energy of the AB interaction was set to zero, while the BB interaction was assumed to be non-repulsive. The grand canonical ensemble Monte Carlo simulation, performed for the particular system with $u_{AB}=u_{BB}=0$, showed that complete wetting occurs at low temperatures, even for very weakly adsorbing surfaces.

In this work, we applied the same lattice model, but for a wider range of parameters representing the interaction energies between the particles. Three series of systems with $u_{AB}=u_{BB}$, with $u_{AB}=0$ and with $u_{BB}=0$ have been considered. It has been shown that depending on the assumed interactions between the particles, the bulk systems order into the superantiferromagnetic (SAF) or into the antiferromagnetic (AF) phases at low temperatures and at sufficiently high densities, leading to the different topologies of the bulk phase diagrams. The knowledge of the bulk phase behavior is a necessary prerequisite for the study of wetting phenomena on solid surfaces. Our study of wetting has shown that the orientation-dependent interactions may lead to the dewetting and the reentrant wetting transitions in the systems characterized by different interparticle interactions and different strengths of the surface potential.

The paper is organized as follows. In the next section, we briefly recall basic information about the model used and the applied Monte Carlo method. A detailed description of the model has been already given in [35]. In Section 3, we discuss the bulk behavior of the model and demonstrate how the assumed interactions between the particles affect the topology of bulk phase diagrams. Then, in Section 4 we discuss the wetting behavior of the already specified three series of systems. This section is divided into two subsections, in which the systems order into the SAF and AF structures in the bulk. Section 5 describes the results of calculations for an idealized model, which demonstrates how the stability of the ordered SAF and AF multilayer films influences the wetting behavior of different systems. The paper is concluded in Section 6, in which we summarize our findings.

## 2. The Model and Methods

**Figure 1:**
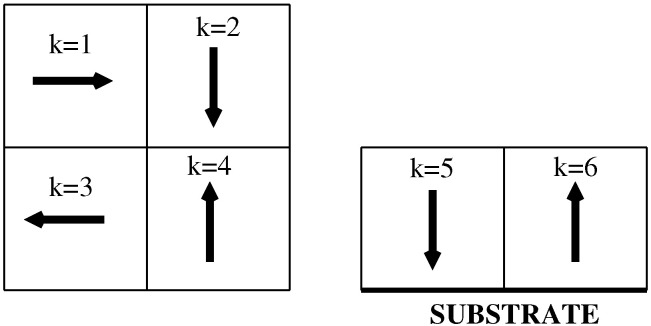
The allowed orientations of particles on the cubic lattice. The left part shows the top view of four “in-plane” orientations, while the right part shows the side view of two “out-of-plane” orientations. The arrows point from part B to part A.

The model used here is essentially the same as described in earlier work [35]. Thus, we consider the particles composed of the two halves, A and B, on a regular cubic lattice and with the interactions limited to the first nearest neighbor. Each particle is assumed to take on one of the six orientations, marked by the parameter k=1,2,…6 (see Figure 1), and the energy of an interaction between a pair of neighboring particles is equal to

(1) $$\begin{array}{ccc} u(k_{i},k_{j},{\vec{r}}_{ij}) & = & w_{AA}\left(k_{i},k_{j},{\vec{r}}_{ij}\right)u_{AA}+w_{AB}\left(k_{i},k_{j},{\vec{r}}_{ij}\right)u_{AB}\\ & + & w_{BB}\left(k_{i},k_{j},{\vec{r}}_{ij}\right)u_{BB} \end{array}$$

In the above, $u_{AA}$, $u_{AB}$ and $u_{BB}$ are the interaction energies corresponding to the relative orientations in which the AA, AB or BB halves face one another, while $w_{AA}\left(k_{i},k_{j},{\vec{r}}_{ij}\right)$, $w_{AB}\left(k_{i},k_{j},{\vec{r}}_{ij}\right)$ and $w_{BB}\left(k_{i},k_{j},{\vec{r}}_{ij}\right)$ are the weights determined by the degrees to which the AA, AB and BB regions overlap and ${\vec{r}}_{ij}$ is the separation vector. In the case of the cubic lattice, the separation vectors are: ${\vec{r}}_{ij}=\left(\pm 1,0,0\right)$, (0,±1,0) and (0,0,±1) and there are seven different values of $u(k_{i},k_{j},{\vec{r}}_{ij})$ (cf. Table 1 of [35]).

Such a lattice fluid is in contact with a solid substrate and the fluid–substrate interaction is described by the potential V(z,k), which depends on the distance from the surface, *z*. When the parts A and B interact differently with the solid substrate, the surface is selective, and the surface potential depends also on the orientation *k* of the fluid particle. Here, we assume that V(z,k) has the following form:

(2) $$V\left(z,k\right)=\left\{\begin{array}{cc} \frac{V_{o,k}}{z^{3}}, & z\leq z_{cut}\\ 0, & z>z_{cut} \end{array}\right.$$

with

(3) $$V_{o,k}=\left\{\begin{array}{cc} 0.5(V_{A}+V_{B}), & k=1,2,3,4\\ V_{A}, & k=5\\ V_{B}, & k=6 \end{array}\right.$$

In the above, $V_{A}$ and $V_{B}$ represent the fluid–solid interaction energies when the particle in the first layer assumes the orientation with its A or B side directed towards the surface (see the right part of Figure 1) and the cut-off distance $z_{cut}$ has been set at z=15.

Since the model has been studied with the Monte Carlo simulation method in the grand canonical ensemble [37,38], only finite systems could be treated. Here we considered systems consisting of $DL^{2}$ sites, arranged in *D* layers of $L^{2}$ sites each. The Hamiltonian of the model reads

(4) $$H=\sum_{\langle i,j\rangle }u\left(k_{i},k_{j},{\vec{r}}_{ij}\right)n_{i}n_{j}+\sum_{l=1}^{D}\sum_{i\in l}V\left(l,k_{i}\right)n_{i}-\mu \sum_{i=1}^{DL^{2}}n_{i}$$

where $n_{i}=1$ or 0, when the *i*-th site is occupied or empty. The first term represents the total energy of the fluid–fluid interaction, with the sum running over all distinct pairs of nearest neighbors, the second term gives the energy of the interaction between the fluid particles and the solid, and μ is the chemical potential.

To study bulk systems, we have mostly used the simulation cells of the size with L=D=20, the periodic boundary conditions applied in all three directions. In some cases, the larger simulation cells with L=D=30 and 40 have been used to see if the finite size effects influence the results. The quantities recorded included the total density

(5) $$\rho_{b}=\frac{1}{DL^{2}}\langle \sum_{i=1}^{DL^{2}}n_{i}\rangle \;,$$

the densities of differently oriented particles

(6) $$\rho_{b,k}=\frac{1}{DL^{2}}\langle \sum_{i=1}^{DL^{2}}n_{i}\delta \left(k_{i}-k\right)\rangle \;,$$

the average potential energy per site

(7) $$\langle u\rangle =\frac{1}{DL^{2}}\langle \sum_{\langle ij\rangle }n_{i}n_{j}u\left(k_{i},k_{j},{\vec{r}}_{ij}\right)\rangle \;,$$

the heat capacity

(8) $$C_{V}=\frac{1}{T^{2}}\left[\langle H^{2}\rangle -{\langle H\rangle }^{2}\right]\;,$$

where $H=H/DL^{2}$, and the density susceptibility

(9) $$\chi_{\rho }=\frac{1}{T}\left[\langle \rho_{b}^{2}\rangle -{\langle \rho_{b}\rangle }^{2}\right]\;.$$

To obtain reliable results at any state point, specified by the temperature and the chemical potential, we performed runs involving $10^{6}$–$10^{7}$ Monte Carlo steps to equilibrate the system, and another $10^{6}$–$10^{7}$ Monte Carlo steps have been used to calculate averages. Each Monte Carlo step consisted of $DL^{2}$ attempts to either create a randomly oriented particle at an also randomly chosen position, or to annihilate one of the randomly chosen particles.

In order to study nonuniform systems, the simulation cell has been assumed to be a slab of fixed width equal to D=80 and with the solid surfaces located at the bottom (z=0) and at the top (z=D+1) of the slab. The linear dimension of each layer was set to L=40, with standard periodic boundary conditions applied to each layer.

The recorded quantities included the local densities of differently oriented particles,

(10) $$\rho_{k}\left(l\right)=L^{-2}\langle \sum_{i\in l}n_{i}\delta \left(k_{i}-k\right)\rangle \;,$$

which allowed calculating the total local density

(11) $$\rho \left(l\right)=\sum_{k=1}^{6}\rho_{k}\left(l\right)\;,$$

the surface excess density

(12) $$\rho_{ex}=\frac{1}{2}\sum_{l=1}^{D}\left(\rho \left(l\right)-\rho_{b}\right)\;,$$

and the surface excesses of differently oriented particles

(13) $$\rho_{ex}\left(k\right)=\frac{1}{2}\sum_{l=1}^{D}\left(\rho_{k}\left(l\right)-\rho_{b,k}\right)\;.$$

The values of $\rho_{b}$ and $\rho_{b,k}$ were obtained by averaging the local densities over the inner layers, with *l* between 21 and 60.

We also calculated the layer energies (per unit surface area), u(l), which were used to calculate the surface excess energy

(14) $$u_{ex}=\frac{1}{2}\sum_{l=1}^{D}\left(u_{l}-u_{b}\right)\;,$$

where $u_{b}$ is the bulk energy (per site), calculated as

(15) $$u_{b}=\frac{1}{40}\sum_{21}^{60}u_{l}\;.$$

One should note that at the low temperatures, the density fluctuations in the bulk phase are considerably smaller than in the surface region. The significant fluctuations in the bulk phase occur only at temperatures close to the critical point. Taking this into account, we used the preferential sampling of the surface region [16,39] to reduce the CPU time. Namely, the first 10 layers and the following 5 layers, adjacent to the surfaces, were sampled 10 and 5 times more frequently than the remaining inner part of the system.

Throughout this work, we have assumed that $u_{AA}=-1.0$ is the unit of energy and hence the energy, temperature and chemical potential were expressed in the reduced units of $|u_{AA}|$. We considered three series of systems, assuming that all interactions are attractive. The first series comprised of systems where $u_{AB}=0$ and $u_{BB}$ was varied. In the second series, we assumed that $u_{AB}=u_{BB}=u^{*}$, and considered different values of $u^{*}$. Finally, in the third series, $u_{BB}$ was set to 0 and $u_{AB}$ was allowed to vary.

The vast majority of results were obtained for non-selective surfaces, i.e., with $V_{A}=V_{B}$. However, we also considered the wetting behavior at selective surfaces, with the positive and negative values of $\Delta V=V_{A}-V_{B}$.

## 3. Bulk Phase Behavior

**Figure 2:**
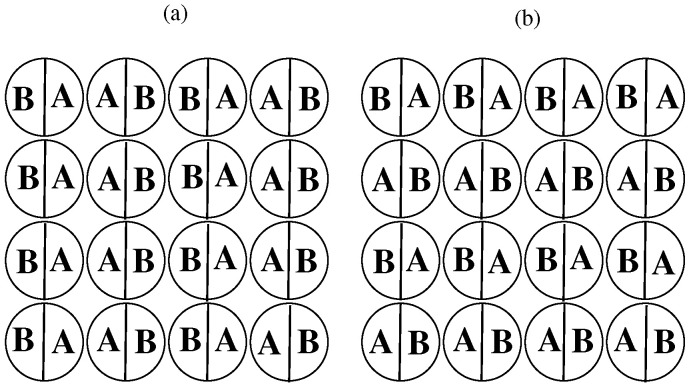
A single plane of the SAF (**a**) and the AF (**b**) phase.

The bulk properties of the series with $u_{AB}=0$ and $u_{BB}\in \left[0,-1\right]$ have already been discussed in [35]. All such systems undergo one first-order transition between the disordered fluid and the orientationally ordered high-density phase at any temperature between zero and the temperature $T_{o}\left(u_{BB}\right)$. At $T_{o}\left(u_{BB}\right)$, the systems of the density ρ=1.0 also undergo the first-order orientational transition between the disordered and the ordered phases. The orientationally ordered phase, of the structure given in Figure 2a exhibits the same ordering as the superantiferromagnetic (SAF) phase of the Ising model [40,41], and the transition between the SAF and paramagnetic (disordered) states is known to be discontinuous [42].

The phase diagrams for any $u_{BB}$ between 0 and −1.0 have the swan neck shape (cf. Figure 12 in [35]). The lack of gas–liquid condensation is caused by the high stability of the orientationally ordered phase. Even in the case $u_{BB}=0$, the AA attraction is sufficient to stabilize the ordered SAF phase over a wide range of temperatures. Thus, these systems exhibit the incipient triple point, resulting from the sufficient lowering of the ordered phase free energy to exclude the presence of a disordered (liquid-like) phase. Such phase behavior was observed in several two- and three-dimensional systems. In particular, in monolayers of nitrogen and krypton on graphite [43] and in the monolayers of noble gases on lamellar dihalides [44]. In those systems, the lack of gas–liquid condensation resulted from the high stability of commensurate phases formed on corrugated surfaces [4,6]. Similarly, the Lennard–Lones fluids confined between crystalline walls [45,46] and non-additive symmetric mixtures [47,48] also demonstrated a lack of gas–liquid condensation.

The coexistence between the dilute and the condensed phases terminates at the temperature $T_{o}\left(u_{BB}\right)$ and ρ=1.0 and $T_{o}\left(u_{BB}\right)$ was found to increase linearly, when the BB attraction becomes stronger, from about 0.404 when $u_{BB}=0$, up to about 0.81 for $u_{BB}=-1.0$.

The systems with $u_{BB}=0$ and those with $u_{AB}=u_{BB}=u^{*}$, also exhibit the first-order orientational order–disorder transition at ρ=1.0, but their phase behavior is qualitatively different. In the case of $u_{BB}=0$, the temperature of the orientational transition at ρ=1.0, $T_{o}\left(u_{AB}\right)$ linearly decreases from about 0.404, when $u_{AB}=0$, to zero, when $u_{AB}=-0.5$ and then, also linearly, increases from zero to about 0.41, when $u_{AB}$ decreases further from −0.5 down to −1.0. This behavior results from the change in the structure of the orientationally ordered phase. As long as $u_{AB}>-0.5$, the ordered phase is the SAF structure, but its stability gradually decreases when the AB attraction increases from $u_{AB}=0$ to $u_{AB}-0.5$. On the other hand, for $u_{AB}<-0.5$, the ordered high-density phase assumes the structure with only AB contacts between the nearest neighbors (see Figure 2b) and corresponds to the antiferromagnetic (AF) ordering in the Ising model [40]. In the particular case of $u_{AB}=-0.5$, the system does not show any orientationally ordered structure. The nature of the ordered phase depends on the sign of the parameter $\epsilon =u_{AA}+u_{BB}-2u_{AB}$. For $u_{AB}>-0.5$, the AA and BB contacts are preferred (ϵ<0), while for $u_{AB}<-0.5$, the AB contacts are preferred (ϵ<0), leading to the AF structure, which also undergoes the first-order orientational order–disorder transition [49].

**Figure 3:**
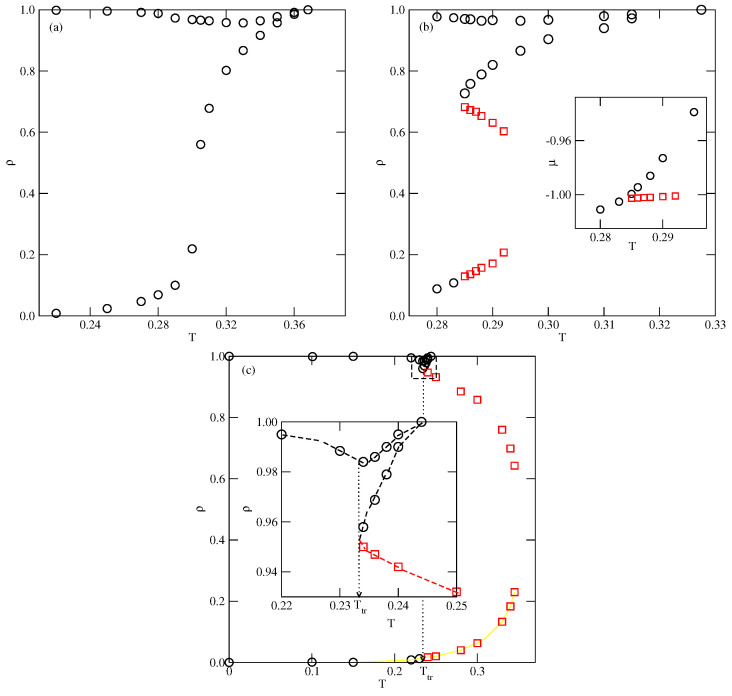
The T−ρ projections of bulk phase diagrams for the systems with $u_{BB}=0$ and $u_{AB}=-0.05$ (**a**), −0.1 (**b**) and −0.2 (**c**). The circles represent the phase boundaries between the disordered and ordered structures, while the squares mark the gas–liquid phase boundaries. The inset to Figure 4b gives the T−μ projection of the phase diagram for the system with $u_{BB}=0$ and $u_{AB}=-0.1$. The inset to Figure 4c shows the region of liquid–solid transition in the enlarged scale.

We have estimated several phase diagrams for the systems with $u_{BB}=0$ assuming different values of $u_{AB}>-0.5$ and Figure 3 shows the examples of phase diagrams for $u_{AB}$ equal to −0.05, −0.1 and −0.2. The phase diagram for $u_{AB}=-0.05$ is qualitatively different from those obtained for lower values of $u_{AB}$ and does not show the gas–liquid condensation. The only transition occurs between the disordered fluid and the orientationally ordered (SAF) phases. Thus, this system shows qualitatively the same phase behavior as those with $u_{AB}=u_{BB}$ (cf. Figure 12 in [35]). The weak AB attraction does not destabilize the SAF phase sufficiently to allow for the disordered liquid to appear. On the other hand, when $u_{AB}=-0.1$ and −0.2, the gas–liquid transition does occur over a certain range of temperatures. Only at sufficiently low temperatures, up to the triple point temperature, $T_{tr}\left(u_{AB}\right)$, does a direct gas–SAF transition take place. At the temperatures above the triple point, a dilute gas phase condenses into an orientationally disordered liquid-like phase and the transition terminates at the critical point. The critical temperature, $T_{c}\left(u_{AB}\right)$, was found to increase when the AB attraction becomes stronger. On the other hand, the triple point temperature as well as $T_{o}\left(u_{AB}\right)$ both decrease when the AB attraction becomes stronger. These changes of $T_{c}\left(u_{AB}\right)$, $T_{tr}\left(u_{AB}\right)$ and $T_{o}\left(u_{AB}\right)$, can be readily explained by taking into account that the lowering of $u_{AB}$ causes the overall attraction in the system to become stronger. Since the gas and liquid phases are orientationally disordered, the critical temperature is proportional to the pair interaction energy averaged over all orientations, which is given by

(16) $$\bar{u}=\frac{1}{36}\sum_{k_{1}=1}^{6}\sum_{k_{2}=1}^{6}u\left(k_{1},k_{2}\right)\;,$$

and hence increases when $u_{AB}$ is lowered from zero to −1.0. In the above equation, we have dropped the dependence of the pair interaction energy $u(k_{1},k_{2})$ on the separation vector, since $\bar{u}$ is the same for any ${\vec{r}}_{ij}$.

Since the stability of the SAF structure is weakened by the AB attraction, both $T_{o}\left(u_{AB}\right)$ and $T_{tr}\left(u_{AB}\right)$ decrease when $u_{AB}$ is lowered. Of course, $T_{tr}\left(u_{AB}\right)$, which has to be lower than $T_{o}\left(u_{AB}\right)$, gradually approaches $T_{o}\left(u_{AB}\right)$ when $u_{AB}$ decreases towards −0.5.

When $u_{AB}<-0.5$, the structure of the orientationally ordered dense phase changes to AF and its stability gradually increases when $u_{AB}$ decreases from −0.5 to −1.0. The calculations have shown the presence of gas–liquid condensation for any $u_{AB}\in \left(-0.5-1.0\right]$. However, we have not been able to reliably estimate the triple point temperatures for the systems with $u_{AB}<-0.5$. This was caused by the fact that the liquid–AF transition occurs when the densities are very close to unity.

Only in the system with $u_{AB}=-1.0$ have we obtained results suggesting that the triple point temperature is slightly lower than $T_{o}\left(u_{AB}=1\right)\approx 0.404$. The calculations performed at T=0.40 have shown that the gas condensed directly into the AF ordered phase of the density about 0.999, while at T=0.402, the gas condensed into the disordered phase of the density about 0.996, which undergoes the transition into the ordered AF phase at the sufficiently high chemical potential. The transition is discontinuous, but with a very small density jump between about 0.9975 and 0.9985. Thus, the triple point is expected to be located at a temperature of about 0.401. When the AB attraction becomes weaker ($u_{AB}>-1.0$), the triple point temperature is expected to be lower and closer $T_{o}\left(u_{AB}\right)$.

**Figure 4:**
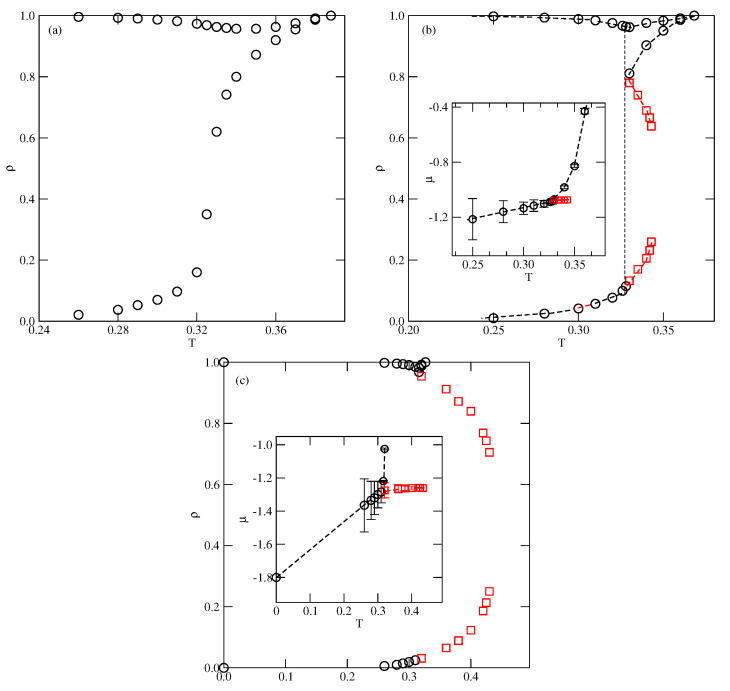
The T−ρ projections of bulk phase diagrams for the systems with $u^{*}=-0.05$ (**a**), −0.1 (**b**) and −0.2 (**c**). The circles represent the phase boundaries between the disordered and ordered structures, while the squares mark the gas–liquid phase boundaries. The insets to (**b**,**c**) give the T−μ projections of phase diagrams.

The systems with $u_{AB}=u_{BB}=u^{*}$ also order into the SAF phase at low temperatures and sufficiently high densities. Their phase behavior is qualitatively the same as in the systems discussed above with $u_{BB}=0$ and $u_{AB}>-0.5$ (see Figure 4). In particular, there is no trace of the gas–liquid transition when $u^{*}=-0.05$. It should be noted that upon the decrease of $u^{*}$ towards −1.0, the systems gradually become more and more similar to the isotropic lattice gas model. This causes the critical temperature of gas–liquid transition to increase when $u^{*}$ decreases. Ultimately, $T_{c}\left(u^{*}=-1.0\right)$ reaches the value of about 1.128, characteristic of the three-dimensional cubic lattice gas model [50]. On the other hand, the triple point temperature and the temperature of orientational order–disorder transition at ρ=1.0 both gradually decrease towards zero when $u^{*}$ approaches −1.0 since there is no orientational order–disorder transition in the case of isotropic interactions.

**Figure 5:**
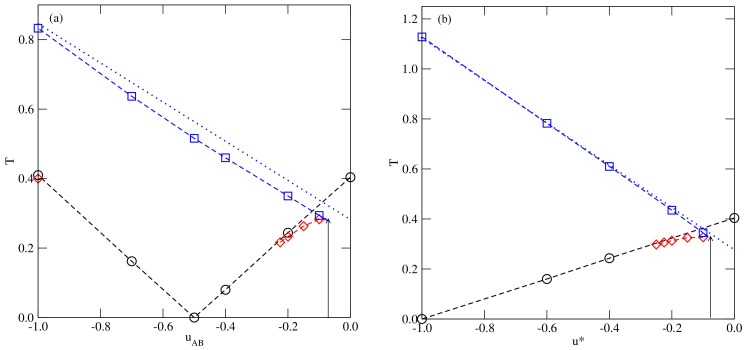
The changes of the critical temperature (squares), the temperature of the orientational order–disorder transition at ρ=1.0 (circles) and the triple point temperature (diamonds) versus $u_{AB}$ for the systems with $u_{BB}=0$ (**a**) and versus $u^{*}$ (**b**). The dotted lines mark the predicted changes of the critical temperature in the systems with isotropic interactions.

Figure 5 summarizes the results for bulk systems belonging to the series with $u_{BB}=0$ and with $u_{AB}=u_{BB}$, and gives the changes of $T_{o}\left(u\right)$, $T_{c}\left(u\right)$ and $T_{tr}\left(u\right)$ with *u* when $u=u_{AB}$ (part a) and $u=u^{*}$ (part b). In both cases, the triple point temperature meets the critical point temperature of the gas–liquid transition when $u_{AB}$ or $u^{*}$ become equal to about −0.08. For the still weaker AB and BB attraction (cf. Figure 3a and Figure 4a) the phase diagrams become qualitatively the same as in the series with $u_{AB}=0.0$.

We have also included in Figure 5 the locations of critical temperatures, $T_{c}\left(\bar{u}\right)$, resulting from the assumption that the pair interaction energy is given by $\bar{u}$, i.e., when the interactions are isotropic. It is evident that $T_{c}\left(u_{AB}\right)$ and $T_{c}\left(u^{*}\right)$ are only slightly lower than $T_{c}\left(\bar{u}\right)$. These results show that close to the critical points liquid is nearly unaffected by the anisotropy of interactions.

## 4. Wetting Behavior

### 4.1. The Systems Ordering into the SAF Phase

#### 4.1.1. The Series with $u_{AB}=0$

From the ground state considerations, it follows [35] that in the case of non-selective walls ($V_{A}=V_{B}=V_{o}$), all systems with $u_{AB}=0$ exhibit complete wetting at T=0 whenever

(17) $$u_{BB}/V_{o}<\sum_{i=1}^{\infty }i^{-3}\approx 1.20206$$

The above inequality implies that the particular system with $u_{BB}=0$ wets any attractive surface at T=0. On the other hand, when the BB interaction becomes attractive, complete wetting occurs only for sufficiently strong surface fields. This agrees with the earlier works devoted to the wetting behavior of systems with isotropic interactions [4,6]. An increase in BB attraction leads to an overall increase in the fluid–fluid attraction and hence may hinder wetting at weakly attractive substrates.

When complete wetting occurs at T=0, the film grows via a series of layering transitions and the sequence of these transitions depends on the magnitudes of $u_{BB}$ and $V_{o}$. In the ground state, the AB contacts are excluded for any $u_{BB}\leq 0$, and therefore only the orientations with AA and BB halves facing each other can appear. Thus, the multilayer films are predicted to have the SAF structure, just the same as the bulk condensed phase.

**Figure 6:**
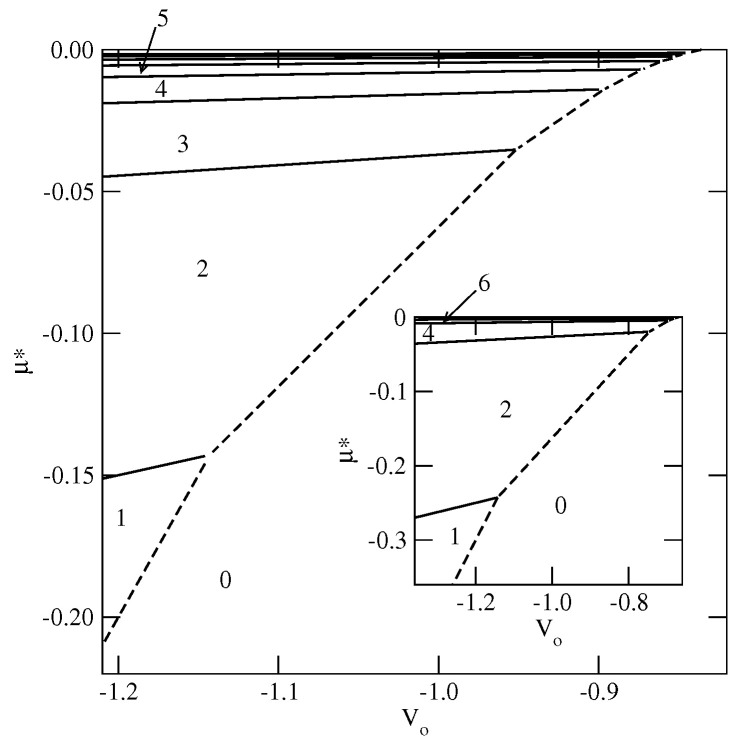
The ground state phase diagrams for $u_{AB}=0$ and $u_{BB}=-1.0$ (the main figure) and for $u_{BB}=-0.8$ (the inset). The regions with different numbers of occupied layers are marked in the figure.

Here, we present the examples of ground state phase diagrams for the systems with $u_{BB}=-1.0$ and −0.8, since these two values of $u_{BB}$ were used in the study of wetting at finite temperatures. The main part of Figure 6 shows the phase diagram at T=0 for $u_{BB}=-1.0$. This system is rather special, since its ground state behavior is the same as in the model with isotropic interactions [9,16]. Note that only the AA and BB contacts are allowed and both are characterized by the same energy. Complete wetting occurs whenever $V_{o}$ is lower than about −0.8319 and the films grow in a layer-by-layer manner. The first layering transition may involve a condensation of one layer, when $V_{o}<-1.0/0.875\approx 1.14286$, or a simultaneous condensation of a larger number of layers when $V_{o}>-1.0/0.875$ and is lower than −0.8319. When $V_{o}\approx -0.8319$ is approached from below, the number of simultaneously condensing layers goes to infinity and occurs right at the bulk coexistence.

The SAF ordering in the film can be realized when the particles in each layer are arranged in rows with alternate orientations with k=1 and k=3, or with k=2 and k=4. Moreover, the films in which the adjacent layers, *i* and i+1, are filled by the particles assuming the orientation with $k_{i}=5\left(6\right)$ and $k_{i+1}=6\left(5\right)$ show the SAF ordering and have the same energy.

The degeneracy of the SAF structure is lifted when the BB attraction becomes weaker, i.e., when $u_{BB}>-1.0$. The inset to Figure 6 gives the ground state phase diagram for $u_{BB}=-0.8$. In this case, the films also grow via a sequence of layering transitions, but each transition involves a simultaneous condensation of two layers. Only when $V_{o}<-1.0/0.875$ foes the first layering transition lead to a monolayer, in which the SAF structure can be realized by different sets of orientations. The formation of the second layer enforces the particles in the first and second layers to assume the orientations with $k_{1}=6$ and $k_{2}=5$, respectively, since the energy of the interlayer interaction, equal to $u_{AA}$, ensures the lowest film energy. Then, the film grows via a series of n→n+2 transitions, with the same sequence of orientations of particles in adjacent layers ($k_{n+1}=6$ and $k_{n+2}=5$).

Any further weakening of the BB attraction does not change this picture and causes only complete wetting to occur at also weaker surface potential, cf. Equation (17).

**Figure 7:**
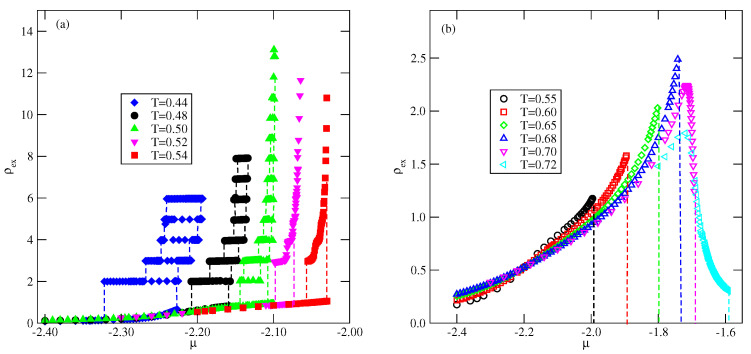
The adsorption–desorption isotherms for the system with $u_{AB}=0$, $u_{BB}=-1.0$ and $V_{o}=-1.0$ at different temperatures (given in the figure), below (**a**) and above (**b**) the dewetting transition temperature.

The first series of calculations at finite temperatures was performed assuming that $u_{BB}=-1.0$ and $V_{o}=-1.0$. Figure 7 presents the adsorption–desorption isotherms recorded at the temperatures between 0.44 and 0.54 (part a) and between 0.55 and 0.72 (part b). At the low temperatures, up to 0.50, the films grow in a layer-by-layer mode and only the first layering transition involves a simultaneous condensation of two layers. This behavior has also been found in the model with isotropic interactions for $V_{o}=-1.0$ [16]. In the finite systems, like those considered here, the first-order transitions are usually accompanied by the pronounced metastability effects [38], which are manifested by the hysteresis loops on the adsorption and desorption branches of isotherms. The precise determination of transition points would require the calculation of free energies of coexisting phases [16]. Taking into account that we have been interested in qualitative behavior only, such calculations were not performed. At the temperatures of 0.52 and 0.54, the remnants of layering transitions are seen only on the desorption branches of isotherms, while the adsorption isotherms suggest the presence of a thin–thick film transition. It should be also noted that the desorption isotherms at T=0.52 and 0.54 do not show the formation of a bilayer film, but three filled layers appear before a final drop to a dilute submonolayer film. The inspection of local density profiles $\rho_{k}\left(z\right)$ demonstrated that multilayer films are built of layers with alternate orientations $k_{n}=6$ and $k_{n+1}=5$ characteristic of the SAF structure and suggest that the system exhibits complete wetting at temperatures up to 0.54.

**Figure 8:**
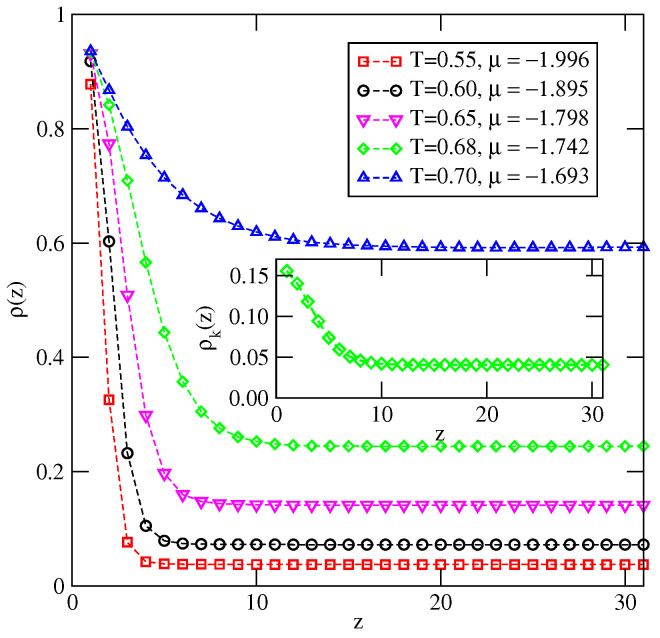
The local density profiles for the system $u_{AB}=0$, $u_{BB}=-1.0$ and $V_{o}=-1.0$, recorded at different temperatures (given in the figure) above the temperature of the dewetting transition and at the chemical potentials close to the bulk coexistence. The inset gives the profiles $\rho_{k}\left(z\right)$ at T=0.68.

When the temperature exceeds 0.54, the behavior of adsorption isotherms changes completely (see Figure 7b). At temperatures between 0.55 and 0.68, the surface excess densities gradually increase, but remain rather low at the bulk coexistence. On the other hand, at still higher temperatures, the surface excess density initially grows and then gradually decays when the chemical potential approaches the bulk coexistence. This is caused by a rather high density of the bulk disordered phase close to the bulk coexistence. Figure 8 presents a series of density profiles recorded at different temperatures and at chemical potentials very close to the bulk coexistence. It is clear that at T≥0.55 the local density smoothly decays towards the bulk density when the distance from the surface increases. The inset to Figure 8 demonstrates that the local densities of differently oriented particles in the film are the same for all orientations. On the other hand, the condensed bulk phase is the orientationally ordered SAF structure at temperatures up to $T_{o}\left(u_{BB}=-1\right)$≈0.81 [35]. Thus, there is a mismatch between the structures of the orientationally disordered films and the ordered bulk phase, which prevents the development of the macroscopically thick (wetting) films and suggests the presence of dewetting transition at temperature $T_{dw}$ between 0.54 and 0.55.

**Figure 9:**
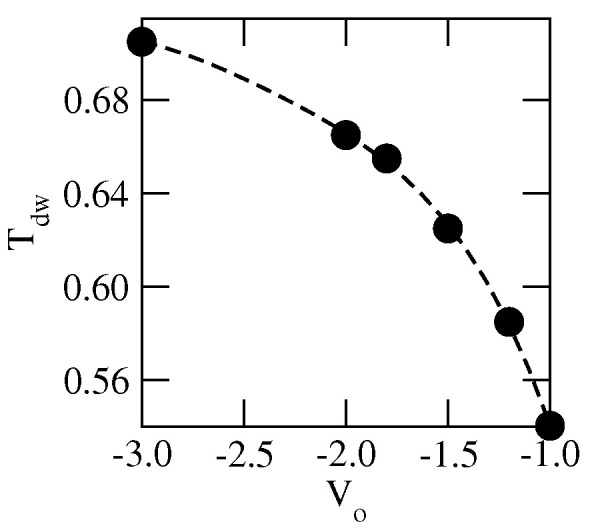
The dewetting transition temperature versus $V_{o}$, for the systems with $u_{AB}=0$ and $u_{BB}=-1.0$.

The stability of the orientationally ordered multilayer films is expected to depend on the strength of the surface potential. An increase of $V_{o}$ causes a stronger pinning of the film to the surface and hence the formation of a thick orientationally ordered adsorbed layer is likely to occur over a wider range of temperatures, suggesting an increase of $T_{dw}$. Indeed, the simulations performed for different $V_{o}$, between −1.0 and −3.0, have shown that $T_{dw}$ increases when the surface potential becomes stronger (see Figure 9). The observed dewetting transition marks the crossover between type-1 and type-2 wetting behaviors. At temperatures above $T_{dw}$, the adsorbed films are quite diffused and the interface between the film and bulk phase becomes broader. Therefore, estimation of the actual film thickness at high temperatures is difficult. The parameter that can be used to measure the film thickness is the average height of the film, 〈h〉, defined in the same way as in the solid-on-solid model [51] by the following equation:

(18) $$\langle h\rangle =L^{-2}\langle \sum_{i=1}^{L^{2}}h_{i}\rangle$$

**Figure 10:**
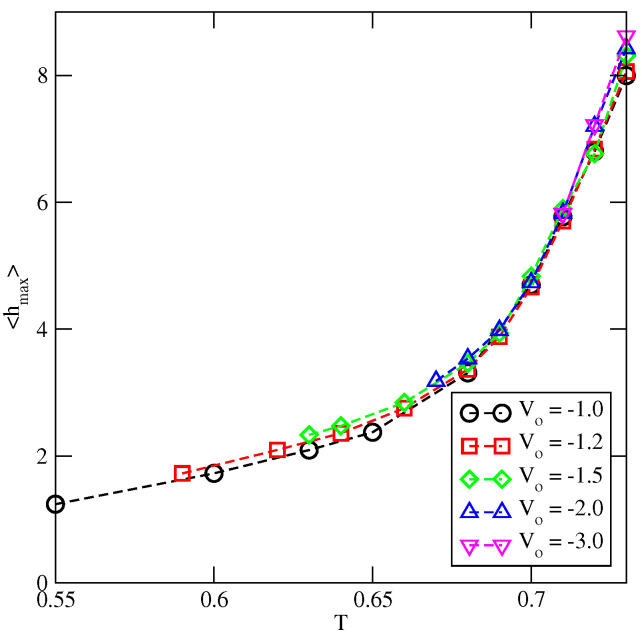
The temperature changes of $h_{max}$ for the systems with $u_{AB}=0$, $u_{BB}=-1.0$ and different $V_{o}$ (given in the figure).

Of course, this parameter underestimates the film thickness at high temperatures, since the film is liquid-like, while 〈h〉 gives the average height of molecular piles without hang-ups. Nevertheless, it provides interesting data about the properties of adsorbed films. At any temperature above the dewetting transition, 〈h〉 is expected to increase with the chemical potential and to reach the finite maximum value, $h_{max}\left(T\right)$, at the bulk coexistence. Figure 10 gives the plots of $h_{max}\left(T\right)$ obtained for the systems characterized by different values of $V_{o}$. Each set of data starts at a different temperature since the dewetting temperature increases when the surface potential becomes stronger. It can be observed that $h_{max}\left(T\right)$ is practically independent of the surface potential strength, at least over the range of $V_{o}$ used. This behavior can be explained by taking into account a rapid decay of the surface potential with the distance from the wall (cf. Equation (2)). An increase of the surface potential strength, from $V_{o}=-1.0$ to $V_{o}=-3.0$, influences the behavior of the adsorbed layer in the region close to the surface only. It has a negligible effect on the upper part of the film and the location of the interface between the film and the bulk. For example, at T=0.7 the parameter $h_{max}$ is equal to about 4.8 and the difference between the fluid–substrate interaction energies of particles in the fifth layer is very small when $V_{o}=-3.0$ and $V_{o}=-1.0$ and is equal to −0.016. Therefore, the film thickness at the bulk coexistence is primarily determined by the incompatibility between the disordered adsorbed film and the ordered bulk condensed phase and is nearly independent of the surface potential strength.

Qualitatively the same behavior was found for different $u_{BB}$ and $V_{o}$. Of course, the location of the dewetting transition depends on the values of $u_{BB}$ and $V_{o}$. For example, when $u_{BB}=-0.2$ and $V_{o}=-0.2$, the dewetting transition occurs at $T_{dw}=0.425\pm 0.005$, and for $u_{BB}=-0.8$ and $V_{o}=-0.8$ it is located at $T_{dw}=0.535\pm 0.005$.

The complete wetting can not be restored at any temperature above the dewetting temperature since the bulk condensed phase remains ordered up to $T_{o}\left(u_{BB}\right)$ and the transition between the disordered and ordered phases is discontinuous. Therefore, the correlation length in the system is finite at any temperature, and the Cahn argument [25] cannot be used here.

Now, we consider the changes in the wetting behavior resulting from the wall selectivity, i.e., when $V_{A}\neq V_{B}$. In such cases, the orientations of particles with their A or B halves directed toward the surface are favored when $\Delta V=V_{A}-V_{B}$ is negative or positive, respectively. Therefore, the structure of multilayer films is expected to depend on ΔV and may affect wetting behavior.

**Figure 11:**
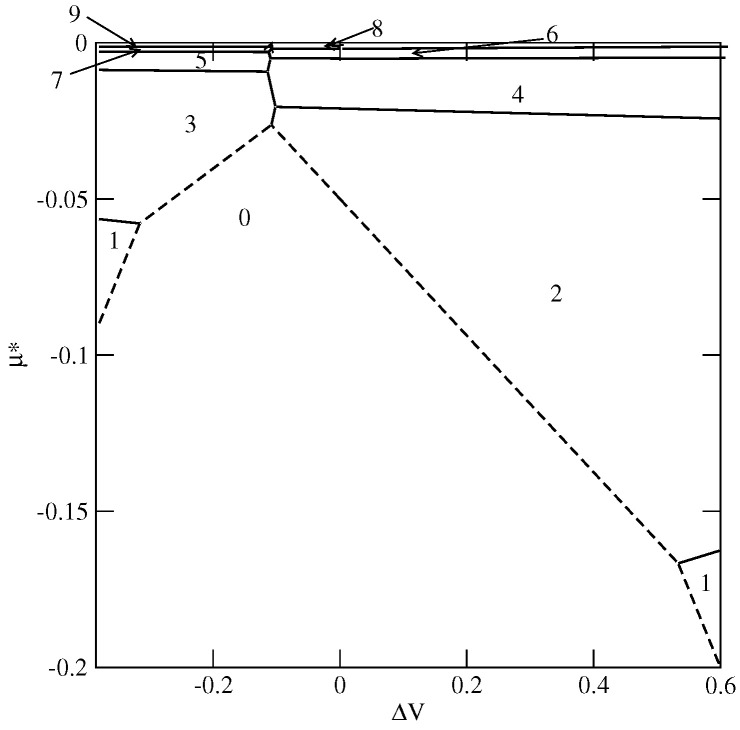
The ground state phase phase diagram for the systems with $u_{AB}=0$, $u_{BB}=-0.8$, $\bar{V}=-0.8$. The regions with different film thicknesses (in filled layers) are marked in the figure by numbers.

Here, we present explicit results for a series of systems with $u_{BB}=-0.8$ and with different values of ΔV, but keeping the average surface potential strength, $\bar{V}=0.5$$\left(V_{A}+V_{B}\right)$ constant and equal to −0.8. This choice of $u_{BB}$ and $\bar{V}$ ensures complete wetting in the ground state. However, the ground state phase diagram (see Figure 11) shows that there are two regions of ΔV with different sequences of layering transitions. When ΔV is lower than about −0.1, only the films with odd numbers of filled layers appear and the orientations of particles in adjacent layers are given by $k_{i}=5$ and $k_{i+1}=6$, for any odd *i*. On the other hand, for ΔV greater than about −0.11, and lower than about 0.533, the films with only even numbers of filled layers are formed. Each layering transition involves a simultaneous condensation of two layers, with alternate orientations $k_{i}=6$ and $k_{i+1}=5$. Only when ΔV becomes higher than about 0.533 does the first layering transition lead to a monolayer film, with $k_{1}=6$, which is followed by the formation of a bilayer, with $k_{2}=5$. The further development of multilayer film occurs via a series of the layering transitions involving a simultaneous filling of two layers, with the alternate orientations of particles in the adjacent layers characterized by $k_{i+1}=6$ and $k_{i+2}=5$. These changes in the ground state behavior result from the enforced changes of particle orientations in neighboring layers. Only over a very narrow range of ΔV around −0.11 may odd and even numbers of occupied layers appear.

**Figure 12:**
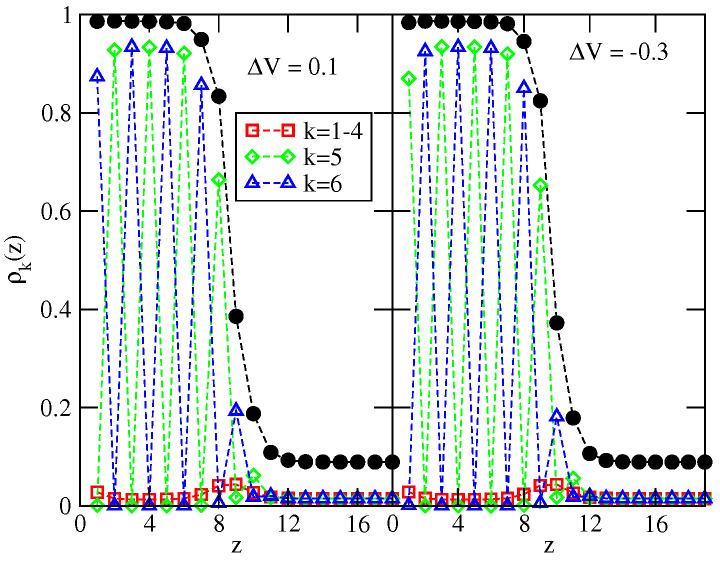
The local density profiles $\rho_{k}\left(z\right)$ for the systems with $u_{BB}=-0.8$, $u_{AB}=0$, $\bar{V}=-0.8$, for two different values of ΔV equal to 0.1 (left panel) and −0.3 (right panel). All profiles were recorded at T=0.56 and μ=−1.69. The filled circles mark the profiles of ρ(z).

**Figure 13:**
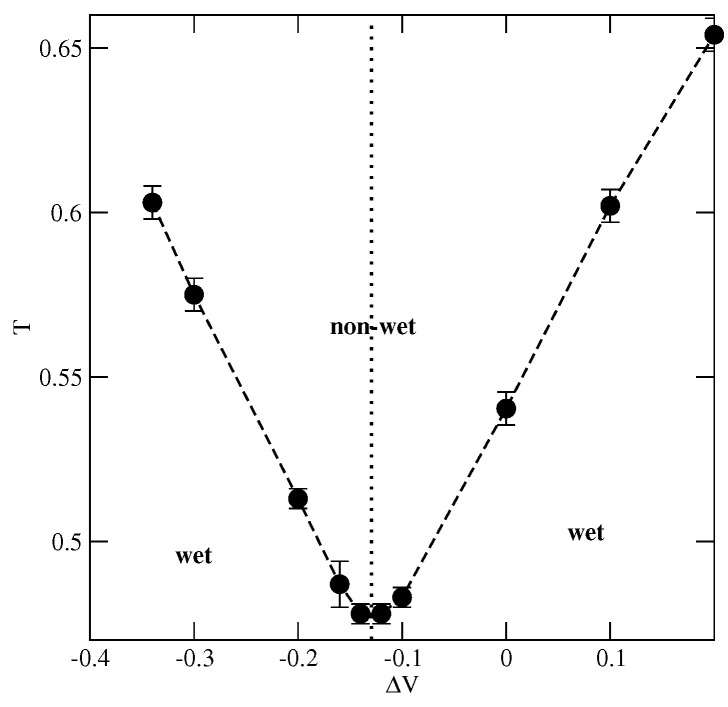
The estimated changes of the dewetting transition temperature with ΔV, for the systems with $u_{AB}=0$, $u_{BB}=-0.8$ and $\bar{V}=-0.8$.

We were interested in the question how wall selectivity influences the location of the expected dewetting transition. To answer this question, a series of simulations for different values of ΔV between −0.34 and 0.2 were performed. The recorded density profiles of differently oriented particles (see Figure 12) confirmed that multilayer films exhibit SAF ordering at low temperatures and the orientations in subsequent layers agree with the ground state predictions. When ΔV=0.1 (ΔV=−0.3), the orientations of neighboring layers are as follows: $k_{i}=6$(5) and $k_{i+1}=5$(6). The stability of SAF ordering in adsorbed films and hence the dewetting temperature, is strongly affected by the wall selectivity. Figure 13 shows that the temperature of the dewetting transition reaches the minimum value for ΔV equal to about −0.13, quite close to the region in which the stacking in subsequent layers changes in the ground state (cf. Figure 11). Then, $T_{dw}$ increases when ΔV becomes higher or lower than about −0.13 due to stronger pinning of adsorbed films to the surface. It does not matter which of the two already mentioned sequences of particle orientations in neighboring layers appear since both lead to the SAF ordering compatible with the structure of the dense bulk phase. In consequence, the changes of $T_{dw}$ are nearly symmetric around the minimum of $T_{dw}$ at ΔV≈−0.13.

#### 4.1.2. The Series with $u_{AB}=u_{BB}$ and the Series with $u_{BB}=0$ and $u_{AB}>-0.5$

It has already been shown in Section 3 that the bulk systems with $u_{AB}=u_{BB}=u^{*}$ and with $u_{BB}=0$ and $u_{AB}>-0.5$ exhibit qualitatively the same phase behavior. In particular, they order into the SAF structure at sufficiently high densities, and at temperatures up to $T_{o}\left(u^{*}\right)$. All these systems exhibit the gas–liquid transition over a certain range of temperatures and the critical temperature linearly increases when $u^{*}$ or $u_{AB}$ decreases. Moreover, the temperatures of orientational order–disorder transition, $T_{o}\left(u^{*}\right)$ and $T_{o}\left(u_{AB}\right)$, decrease linearly from about 0.404 to zero when $u^{*}$ approaches −1.0, or $u_{AB}$ approaches −0.5 (cf. Figure 5).

From the ground state calculations, it follows that in non-uniform systems the behavior of systems with $u_{AB}=u_{BB}=0$ and those with $u_{BB}=0$ and $u_{AB}>-0.5$ is also qualitatively the same since the AB contacts do not appear. Thus, the formation of multilayer films usually involves n→n+2 layering transitions. Only when $V_{o}<-1/0.875$ can the monolayer films appear. Then, the condensation of the second layer takes place. Further growth of the film occurs via a series of n→n+2 transitions.

Below, we present the results which demonstrate that the wetting behavior of these two series is very similar.

We begin the discussion by considering the series with $u_{AB}=u_{BB}$. The majority of calculations were performed for $u^{*}=-0.2$. The bulk system with $u^{*}=-0.2$ is characterized by $T_{c}\left(u^{*}=-0.2\right)\approx 0.435$, $T_{o}\left(u^{*}=-0.2\right)\approx 0.327$ and $T_{tr}\left(u^{*}=-0.2\right)$≈0.316. The surface potential strength varied between −0.15 and −0.8. One should note that for this choice of $u^{*}$, complete wetting at T=0 requires $V_{o}$ to be lower than about −0.1664.

**Figure 14:**
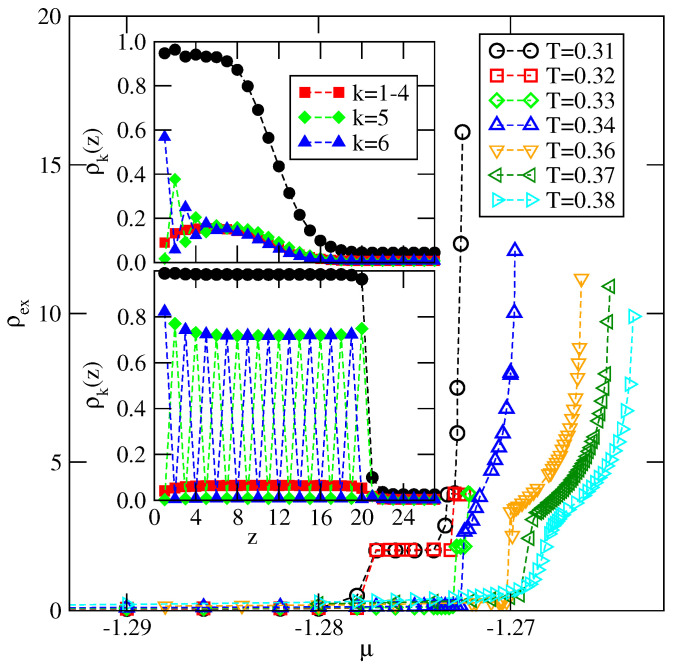
The main part shows adsorption isotherms for the system with $u^{*}=-0.2$ and $V_{o}=-0.22$, recorded at different temperatures (given in the figure). The lower and upper insets show the density profiles ρ(z) (filled circles) and $\rho_{k}\left(z\right)$ (open symbols) recorded at T=0.31 and μ=−1.2877 and at T=0.34 and μ=−1.2698, respectively.

Figure 14 presents the examples of adsorption isotherms obtained for $V_{o}=-0.22$ and at the different temperatures between 0.31 and 0.38. At temperature T=0.31, which is lower than the bulk triple point temperature, the film grows via a sequence of n→n+2 transitions and reaches a large thickness. The lower inset in Figure 14 shows the plots of ρ(z) and $\rho_{k}\left(z\right)$ recorded at T=0.31 and the chemical potential close to the bulk coexistence. These profiles demonstrate the SAF ordering in the film and hence we conclude that complete wetting takes place. On the other hand, at T=0.32 and 0.33, i.e., slightly above the triple point temperature, only four solid-like layers appear at the bulk coexistence and hence only partial wetting occurs. However, a further increase in temperature to 0.34 and above leads again to the development of quite thick films. The upper inset to Figure 14 gives the profiles of ρ(z) and $\rho_{k}\left(z\right)$ at T=0.34, which are quite different from those recorded at T=0.31. Only in the first few layers are the alternate orientations, with k=6 and 5, still favored. At larger distances from the surface, all orientations become very similar, indicating that the thick films are orientationally disordered, exactly as in the bulk liquid phase. From the above results, it follows that there is a dewetting transition, which occurs right at the bulk triple point temperature, and the reentrant wetting transition at a certain temperature $T_{rw}$, above the triple point, since the films and the bulk are liquid-like. One should note that at *T* between 0.34 and 0.36 the first-order prewetting transition appears, implying the presence of a first-order wetting transition.

The temperature of the reentrant wetting transition is dominated by the stability of the orientationally ordered films since complete wetting is possible only when the adsorbed phase is orientationally disordered. It was shown in Section 4.1.1 that in the case of $u_{AB}=0$, the stability of the orientationally ordered adsorbed films increases when the surface potential becomes stronger.

**Figure 15:**
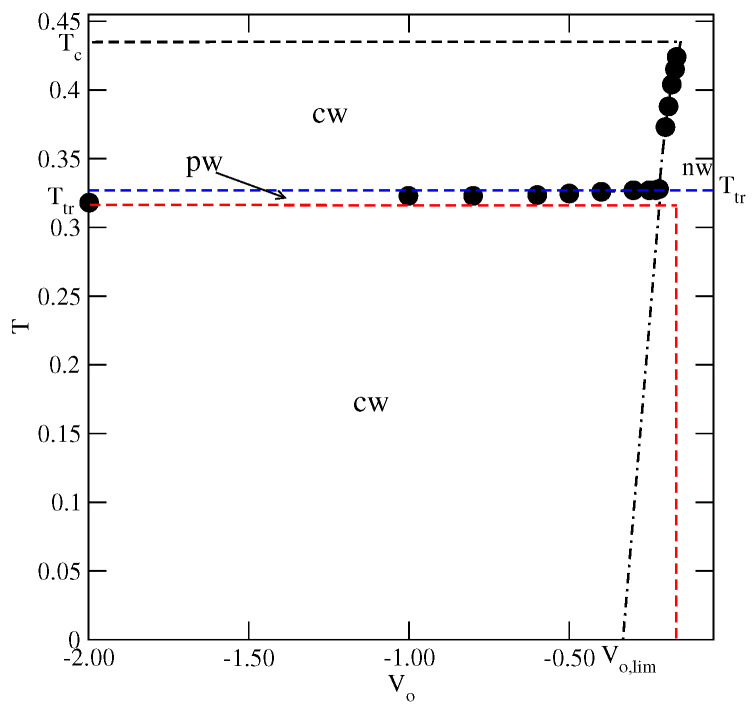
The regions of complete (cw) and partial (pw) wetting for the systems with $u^{*}=-0.2$. The horizontal dashed lines mark $T_{tr}\left(u^{*}=-0.2\right)$, $T_{o}\left(u^{*}=-0.2\right)$ and $T_{}\left(u^{*}=-0.2\right)$ of the bulk system. The vertical dashed line marks the value of $V_{o}$ which delimits the regions of the non-wetting and complete wetting in the ground state. The dash-dotted line marks the expected locations of the wetting temperature in isotropic systems with $\bar{u}=-0.4$.

**Figure 16:**
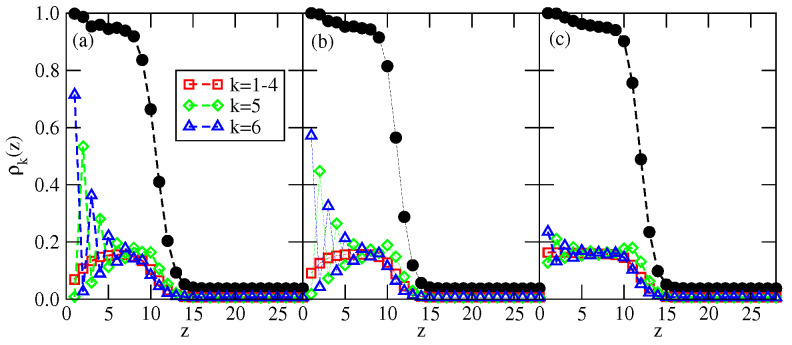
The local density profiles recorded for $u^{*}=-0.2$, at T=0.33 and μ=−1.2725, for three different values of $V_{o}=-1.0$ (**a**), −4.0 (**b**) and −8.0 (**c**). The filled circles correspond to ρ(z), while the open symbols correspond to $\rho_{k}\left(z\right)$.

The results of the simulations carried out for $u^{*}=-0.2$ and the different values of $V_{o}$ have shown, however, that $T_{rw}$ decreases slightly from about 0.327 when $V_{o}=-0.22$, to T≈0.322, when $V_{o}=-2.0$ (see Figure 15). At temperatures between $T_{tr}$ and $T_{rw}$, the films are rather thin and orientationally ordered, though they attain a gradually increasing thickness at the bulk coexistence when the strength of surface potential increases. For example, in the systems with $V_{o}=-0.8$, −1.0 and −2.0, the adsorbed films consist of four, six and eight filled layers, respectively. At temperatures above $T_{rw}$, only the layers adjacent to the surface show remnants of orientational ordering, while the rest of the film is orientationally disordered. This is illustrated in Figure 16, which presents the local density profiles, ρ(z) and $\rho_{k}\left(z\right)$, recorded at the same temperature, T=0.33, and the chemical potential μ=−1.272, but for different values of $V_{o}$ equal to −1.0, −4.0 and −8.0. The film thickness increases only slightly when the surface potential becomes stronger, while its structure changes considerably and becomes increasingly similar to the bulk disordered phase, due to the suppression of the orientational ordering in the layers adjacent to the surface.

The wetting mode changes for the weakly attractive surface potentials with $V_{o}>-0.22$. Of course, complete wetting occurs at low temperatures, up to $T_{tr}\left(u^{*}=-0.2\right)$, but only some very dilute submonolayer films were found at temperatures between $T_{tr}\left(u^{*}=-0.2\right)$ and the temperature of the reentrant wetting transition. Figure 16 shows that the reentrant wetting temperatures for systems with $V_{o}$ between −0.22 and −0.16 increase quite rapidly and rise to the bulk critical point when $V_{o}\approx -0.155$.

This behavior can be explained by taking into account that reentrant wetting may occur only when the growing film is orientationally disordered. In such films, all orientations are nearly equally probable and hence, the energy of the fluid–fluid interaction is close to $\bar{u}$, being equal to −0.4 when $u^{*}=-0.2$. Therefore, the possibility of wetting is governed by the ratio $\bar{u}/V_{o}$ [9] and complete wetting at T=0 takes place only when $V_{o}$ is lower than $V_{o,lim}\approx -0.3328$. Therefore, for the surface potential with $V_{o}>V_{o,lim}$, complete wetting occurs at the finite temperatures only, and the wetting temperature is expected to gradually increase towards the critical point when the surface potential becomes weaker. Below the wetting temperature, the adsorbed films should remain very thin and exhibit the prewetting transition at the temperatures above the wetting temperature, in agreement with our results. The results given in Figure 15 demonstrate that for the systems with $V_{o}>-0.22$, the temperature of the reentrant wetting transition can be smoothly extrapolated to zero when $V_{o}$ approaches $V_{o,lim}$ from above.

The above scenario holds for $V_{o}<-0.166$, since the orientationally ordered films are not stable for the weaker surface potential. Therefore, such systems do not show the triple point dewetting transition and complete wetting occurs only at sufficiently high temperatures.

**Figure 17:**
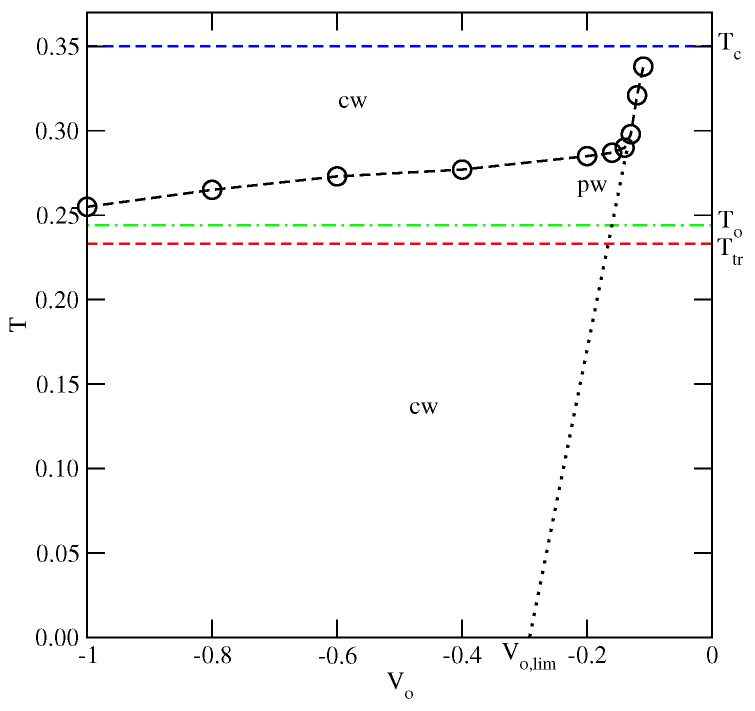
The regions of the complete (cw) and partial (pw) wetting for the systems with $u_{AB}=-0.2$. The horizontal dashed lines mark the triple point, the critical point and the order–disorder transition temperatures of the bulk system. The dotted line marks the expected locations of the wetting temperature in isotropic systems with $\bar{u}=-0.35$.

Now, we turn to the series with $u_{BB}=0$ and $u_{AB}>-0.5$ and present the results obtained for the system with $u_{AB}=-0.2$, in which $T_{tr}\left(u_{AB}=-0.2\right)\approx 0.233$, $T_{c}\left(u_{AB}=-0.2\right)\approx 0.35$ and $T_{o}\left(u_{AB}=-0.2\right)\approx 0.244$. We have chosen this particular system because its triple point temperature is sufficiently high to allow for efficient use of Monte Carlo simulation. Despite some rather large metastability effects, we were able to observe the layering transitions leading to the thick orientationally ordered films at the temperatures below the triple point. Some orientationally ordered films have also been observed over a certain range of temperatures above the triple point, but with the thickness limited to a few occupied layers only. At the still higher temperatures, but below the bulk critical point, the films were found to lose the orientational ordering and the thick adsorbed layers were observed to develop again. Thus, there is a dewetting transition at the triple point and a reentrant wetting transition above the triple point. The results of the calculations carried out for the different values of $V_{o}$ are summarized in Figure 17 and show qualitatively the same behavior as in the series with $u_{AB}=u_{BB}$ (cf. Figure 15).

In particular, for $V_{o}$ lower than about −0.16, the temperature of the reentrant wetting transition gradually decays when the surface potential becomes stronger. Surprisingly, the temperature of the reentrant wetting transition exceeds $T_{o}\left(u_{AB}=-0.2\right)$, unlike in the systems with $u_{AB}=u_{BB}$. In general, the stability of the orientationally ordered films in the systems with $u_{BB}=0$ is lower than in the systems with $u_{AB}=u_{BB}$ due to the lack of BB attraction. This is reflected by the considerably lower triple point and the orientational order–disorder transition temperatures in the series with $u_{BB}=0$. It should be noted, however, that the reentrant wetting temperatures in the system with $u_{BB}=0.0$ and $u_{AB}=-0.2$ are considerably lower than in the case of $u^{*}=-0.2$.

In the region of weak surface potentials, with $V_{o}>-0.16$, the wetting behavior is governed by the ratio $\bar{u}/V_{o}$ and hence the temperature of rewetting transition increases quite rapidly and approaches the bulk critical point for $V_{o}\approx -0.104$. Again, the results are qualitatively quite similar to those obtained for $u^{*}=-0.2$ (cf. Figure 15).

### 4.2. The Systems Ordering into AF Structure

Finally, we turn to the systems with $u_{AB}<-0.5$, which order into the AF phase in the bulk. In the ground state, the adsorbed films also order into the AF structure and the adsorbed films grow in a layer-by-layer mode. Of course, the first layering transition may involve a simultaneous condensation of a larger number of layers when the strength of surface potential is reduced. At T=0, complete wetting occurs whenever

(19) $$\frac{u_{AB}}{V_{o}}<\sum_{i=1}^{\infty }i^{-3}\approx 1.20206.$$

We have already shown that the bulk triple point temperature $T_{tr}\left(u_{AB}\right)$ increases when the strength of the AB attraction increases and is only slightly lower than the orientational order–disorder transition temperature in the close-packed system, $T_{o}\left(u_{AB}\right)$. In particular, when $u_{AB}=-1.0$, we found that $T_{tr}\left(u_{AB}=-1.0\right)\approx 0.4$ and $T_{o}\left(u_{AB}=-1.0\right)\approx 0.41$. The critical temperature of the gas–liquid transition in this system is equal to $T_{c}\left(u_{AB}=-1.0\right)\approx 0.833$.

**Figure 18:**
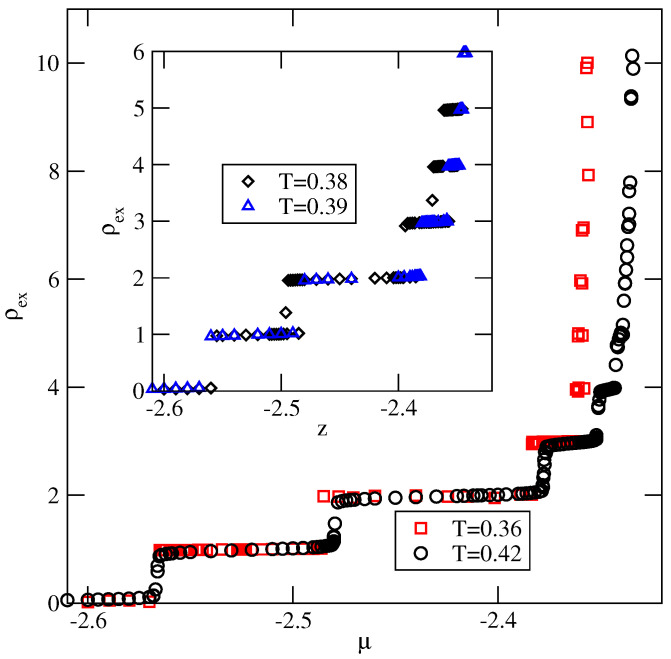
The adsorption isotherms for the system with $u_{AB}=-1.0$ and $V_{o}=-1.00$. The main part shows the isotherms at the temperatures, at which complete wetting occurs, while the isotherms presented in the inset correspond to the temperature region over which only a partial wetting appears.

The adsorption isotherms recorded for the system with $u_{AB}=-1.0$ (see Figure 18) have shown the formation of the thick films via a series of the n→n+1 layering transitions, at temperatures lower than 0.37. Then, at temperatures between 0.37 and 0.39, the adsorbed films of only finite thickness appear (see the inset to Figure 18). At the temperatures above the bulk triple point, the adsorbed films still grow in a layer-by-layer mode, and attain large thickness, as shown by the isotherm at T=0.42.

**Figure 19:**
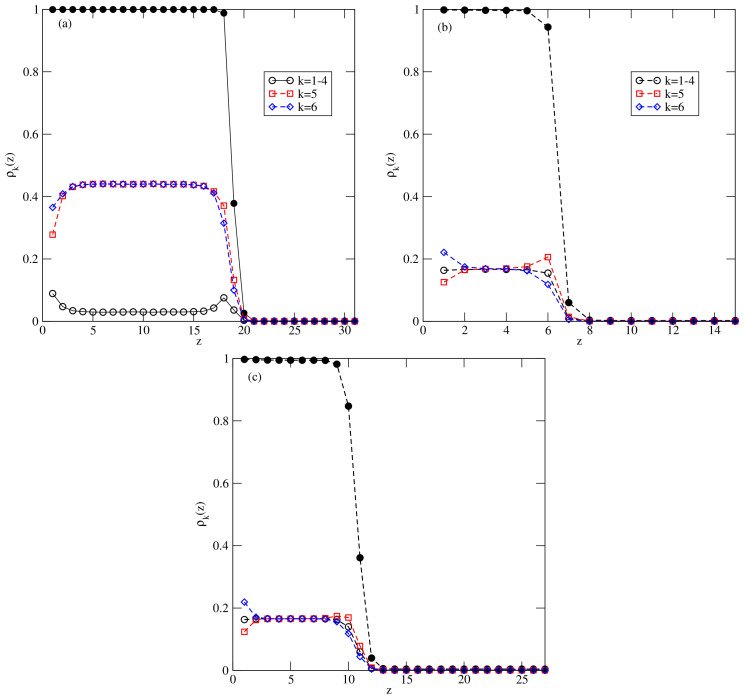
The local density profiles recorded for $u_{AB}=-1.0$, $u_{BB}=0$ and $V_{o}=-1.0$, recorded at T=0.36 and =−2.359 (**a**), T=0.39 and =−2.341 (**b**) and at T=0.42 and =−2.3345 (**c**). The filled circles correspond to the total local density profile, ρ(z).

A sudden drop of the film thickness close to the bulk coexistence occurring at the temperatures between 0.36 and 0.37 is a consequence of the structural changes in adsorbed films. Figure 19 presents the local density profiles, ρ(z) and $\rho_{k}\left(z\right)$, for multilayer film, recorded at T=0.36 (part a), at T=0.39 (part b) and at T=0.42 (part c).

The profiles at T=0.36 show a quite well developed AF structure in the film, in which the particles predominantly assume orientations with k=5 and 6 in every layer. On the other hand, at T=0.39, the film consisting of six occupied layers is orientationally disordered and quite similar behavior has been observed at T=0.37 and 0.38. Of course, at the temperatures exceeding the triple point temperature, the films remain disordered, and attains a large thickness close to the bulk coexistence (see Figure 19c).

The above results show that there is a dewetting transition at the temperature between 0.36 and 0.37. At any temperature up to the triple point at $T_{tr}\approx 0.4$, the bulk condensed phase has the ordered AF structure, while the film already becomes disordered at T=0.37. At temperatures above the triple point, the bulk condensed phase and the adsorbed films are both disordered. Therefore, there is a reentrant wetting transition right at the bulk triple point temperature.

Triple point wetting is a well known phenomenon, observed in many real systems [8,19,23,24,52,53,54]. It has been suggested that triple point wetting results from the mismatch between the lattice constants of solid adsorbed and bulk phases [8,54]. Gittes and Schick [22] applied linear elasticity theory to adsorbed films at T=0 and demonstrated that a strain may lead to a long-range force that considerably inhibits wetting.

In the lattice model considered presently, there is no mismatch between the lattice constants of surface and bulk phases, and hence the mechanism of the dewetting transition below the triple point temperature and the reentrant triple point wetting is bound to be different than in the already mentioned systems, which show the triple point wetting transition.

The main factor leading to the dewetting transition below the triple point temperature is the high fragility of the AF structure, as compared with the SAF structure.

For example, temperatures of the order–disorder transition in the systems with $u_{AB}=u_{BB}=0$ and with $u_{BB}=0$ and $u_{AB}=-1.0$ are very similar and equal to about 0.41 and 0.404, respectively (cf. Figure 5a). However, in the case of $u_{AB}=u_{BB}=0$, the SAF phase is so stable that it excludes the appearance of a liquid phase. It was shown in [35] that the multilayer films have a quite well-developed SAF structure, even at temperatures exceeding the critical point of layering transitions. In fact, even at T=0.4, i.e., in close vicinity to the bulk order–disorder transition, the bilayer film formed on the surface with $V_{o}=-1.0$ was found to form a quite well-ordered SAF structure. On the other hand, the adsorption isotherms, given in Figure 18 and the density profiles, given in Figure 19b, show that in the system with $u_{BB}=0$, $u_{AB}=-1.0$ and $V_{o}=-1.0$, the film with six occupied layers disorders below the critical temperatures of the layering transitions.

In the case of the system with $V_{o}=-0.85$, which also exhibits complete wetting in the ground state, we have found similar behavior with a lower temperature of the dewetting transition, located between 0.34 and 0.35. The reentrant complete wetting occurs at the triple point temperature again. The temperature of the dewetting transition has to go to zero when $V_{o}$ approaches the limiting value of about −0832 from below.

**Figure 20:**
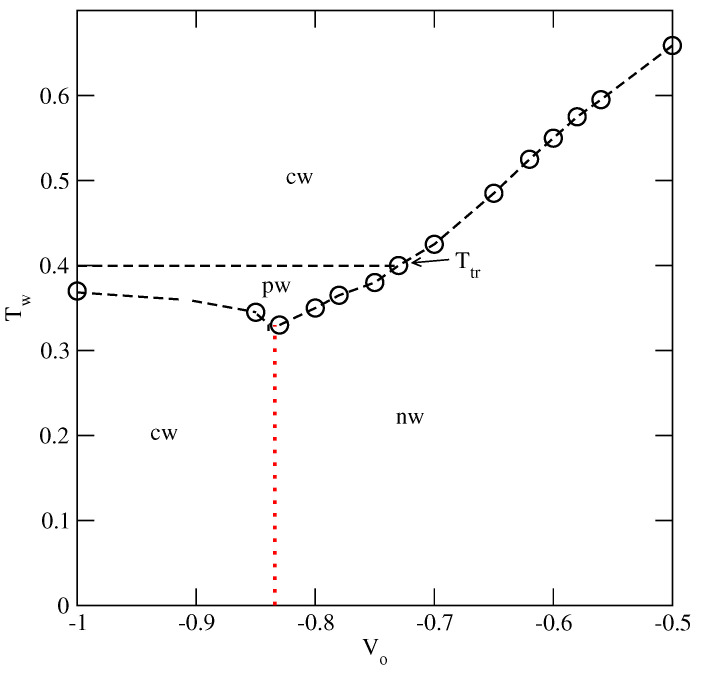
The regions of complete wetting (cw), the partial wetting (pw) and the non-wetting (nw) for the systems with $u_{AB}=-1.0$. The horizontal dashed line marks the triple point temperature of the bulk system. The vertical dashed line marks the value of $V_{o,lim}$, which delimits the non-wetting and complete wetting regions in the ground state. The dotted vertical line marks the expected value of $V_{o}$, which delimits the non-wetting and complete wetting regions at T=0 in the isotropic systems with $\bar{u}=-0.75$.

The wetting behavior has to change when $V_{o}>-0.832$, since complete wetting can take place at finite temperatures only. Indeed, such systems were found to exhibit non-wetting behavior at low temperatures, manifested by some very small surface excess densities along the adsorption isotherms, up to the bulk condensation. However, complete wetting can be expected to occur at a certain wetting temperature, $T_{w}$, which depends on the strength of the surface potential. We performed simulations aiming at the estimation of the wetting temperatures in a series of systems with $V_{o}$, between −0.83 and −0.50 and the results are summarized in Figure 20. For $V_{o}$ lower than about −0.73, we found a crossover between the non-wetting and the partial wetting regimes at temperatures below the bulk triple point and the complete wetting right at the triple point, just as in the systems with $V_{o}<-0.832$. The temperature of the crossover between the non-wetting and the partial wetting increases when the surface field becomes weaker and reaches the temperature of the bulk triple point for $V_{o}\approx -0.73$.

**Figure 21:**
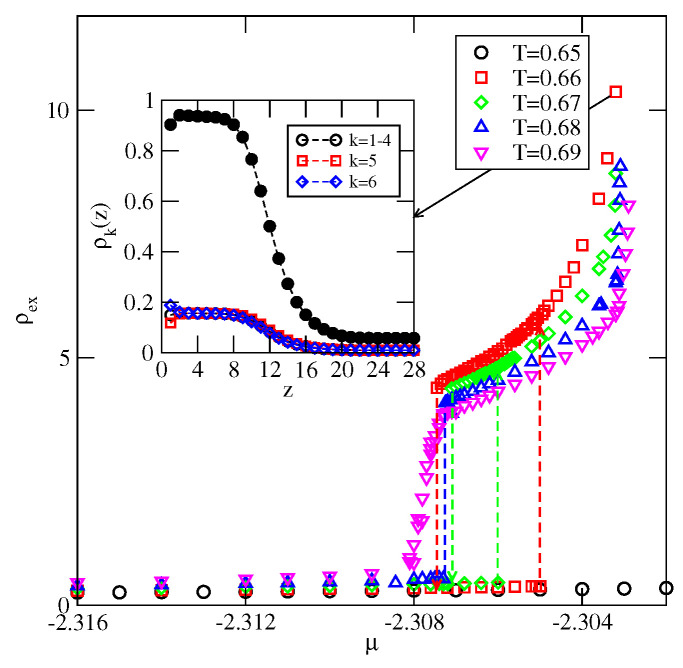
The main figure shows a series of adsorption isotherms for the system with $u_{AB}=-1.0$ and $V_{o}=-0.5$. The inset presents the local density profiles recorded at T=0.66 and μ=−2.3032. The filled circles mark the total local density, ρ(z), while the open symbols correspond to $\rho_{k}\left(z\right)$.

For $V_{o}$ greater than about −0.73, the recorded adsorption isotherms have shown that below the wetting temperature only the dilute submonolayer films appeared (non-wetting regime), below the wetting transition temperature. Figure 20 shows that the wetting temperature increases when the surface potential becomes weaker. Figure 21 shows the examples of adsorption isotherms for the system with $V_{o}=-0.50$, recorded at different temperatures between T=0.65 and 0.69. At T=0.65, the adsorption remains very low upon the approach to the bulk coexistence, implying that the system is a non-wet region. At temperatures between 0.66 and 0.68, the first-order prewetting transition occurs and thick films are orientationally disordered (see the inset to Figure 21). The isotherms also show that the critical temperature of the prewetting transition is located between 0.68 and 0.69. The presence of the prewetting transition is specific to the systems undergoing the first-order wetting transition [3].

## 5. The Stability of Ordered Films and Wetting Behavior

Let us consider an idealized lattice model in which the adsorbed film consists of a certain number, *l*, of filled layers and assume that only the orientations of particles are allowed to change. Such a model allows us to verify the predictions stemming from the ground state calculations and to investigate how the stability of ordered films changes with temperature. We calculated the temperature changes of the potential energy, the heat capacity and the density profiles of differently oriented particles in the films with different numbers of occupied layers and ordering into the SAF and the AF structures. All such systems are effectively two-dimensional and one may expect that the effects of statistical fluctuations are more pronounced than in three dimensions, leading to the lowering of the order–disorder transition temperature.

Here, we present the results for two systems, with $u_{AB}=0$ and with $u_{BB}$ equal to either 0.0 or −1.0. In the bulk, the temperatures of the order–disorder transition of these two systems are nearly the same and are equal to $T_{o}\left(u_{BB}=0\right)\approx 0.404$ and $T_{o}\left(u_{BB}=1-0\right)$≈0.41, but order into the SAF or AF structure, respectively.

**Figure 22:**
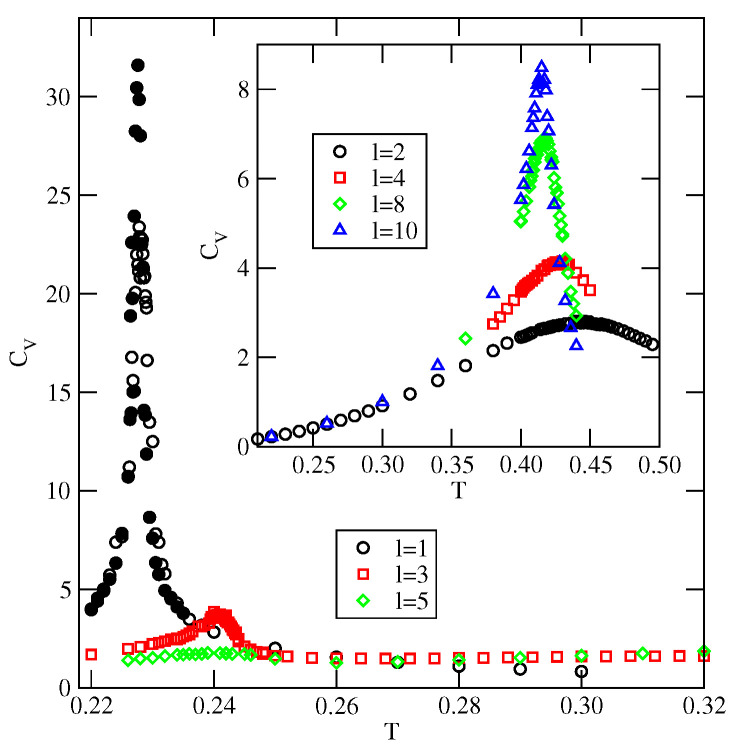
The heat capacity curves for the system with $u_{BB}=0$ and $u_{AB}=0.0$. The main part (inset) shows the results for odd (even) numbers of occupied layers. The open and filled symbols mark the results of the calculations for the simulation cells with L=40 and 60, respectively.

Figure 22 gives a series of heat capacity curves for $u_{BB}=0.0$ and different numbers of occupied layers. The second-order transitions occur in the strictly two-dimensional monolayer as expected. It should be noted that an increase of the simulation cell leads to a shift of the temperature, at which the heat capacity reaches a maximum and then the height of the maximum increases, as predicted by the finite size scaling theory of second-order transitions [55]. The stable monolayer appears only when the surface potential is stronger than 1/0.875, while the formation of stable films with any other odd number of layers is not possible at all. The films with even numbers of occupied layers are stable at low temperatures for any $V_{o},<0$ and disorder continuously over a wide range of temperatures (see the inset to Figure 22). The heat capacity does not exhibit any anomalies, but only some broad maxima at the temperatures above $T_{o}(u_{BB}=0$. These maxima gradually become sharper and are shifted toward $T_{o}(u_{BB}=0$ when the film thickness increases. The SAF ordering in the films of finite thickness correspond to a stacking of the bilayer lamellae with the fixed and the same orientations of particles in each lamella. At very low temperatures, the ordered structure is non-generated and hence its entropy is zero. When the temperature increases, thermal fluctuations gradually lead to some changes in the particle orientations.

We performed similar calculations for other systems ordering into the SAF structure and the results were qualitatively the same. In all cases, the disordering of the multilayer films was found to be gradual.

The model overestimates the stability of multilayer films since it neglects the density fluctuations, due to the promotion of particles to higher layers and possible desorption. On the other hand, it does not take into account the effect of surface potential on the film properties. It was shown in Figure 9 that the stability of the SAF structure in the adsorbed films in the series with $u_{AB}=0$ may considerably increase when the surface potential becomes stronger, increasing the dewetting temperature. However, in the series with $u_{BB}=0$ and ${}_{AB}>-0.5$ and with $u_{AB}=u_{BB}$, we found that the dewetting temperature slightly decreases when the surface potential becomes stronger (cf. Figure 15 and Figure 17). Nonetheless, in both cases, the stability of the ordered adsorbed films is enhanced in comparison with the bulk and the dewetting occurs at temperatures above the bulk triple point temperature.

**Figure 23:**
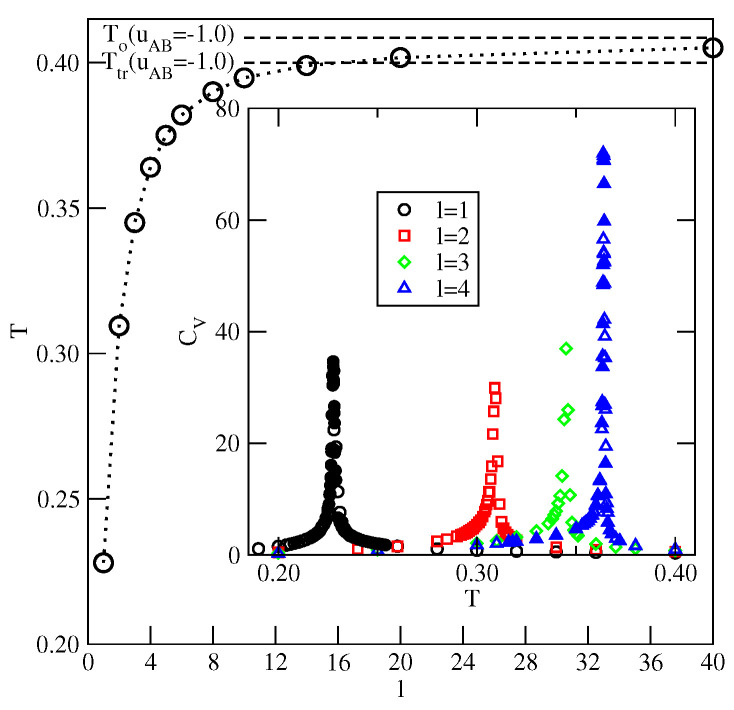
The main part shows the changes of the order–disorder transition temperature versus the number of fully occupied layers in the system with $u_{BB}=0$ and $u_{AB}=-1.0$. Horizontal dashed lines mark the locations of $T_{tr}\left(u_{AB}=-1.0\right)$ and $T_{o}\left(u_{AB}=-1.0\right)$. The inset gives examples of the heat capacity curves for the film of different thickness. The open and filled symbols correspond to the results for the simulation cells with L=40 and 60, respectively.

Similar calculations were carried out for the system with $u_{BB}=0$ and $u_{AB}=-1.0$, which orders into the AF structure at sufficiently low temperatures. The inset to Figure 23 presents examples of the heat capacity curves for the films of different thicknesses. The heat capacities exhibit sharp maxima at the gradually increasing temperatures when the film thickness becomes higher. In addition, the heights of those maxima increase when the size of the simulation cell in *x* and *y* directions becomes larger, indicating the presence of continuous phase transitions. In this effectively two-dimensional model, the order–disorder transition of the AF phase is continuous [40]. The main part of Figure 23 shows that the transition temperature increases from about 0.228 when l=1 to about 0.404 for l=40 and is expected to reach $T_{o}\left(u_{AB}=-1.0\right)$ when the number of occupied layers goes to infinity. From the results given in Figure 23, it follows that at a given temperature the formation of ordered AF structure requires the film to reach a certain thickness. Only at sufficiently low temperatures, below the temperature of the order–disorder transition in the film with *l* occupied layers, does the AF structure becomes stable and allows for the formation of the thick ordered adsorbed phase and complete wetting. At higher temperatures, this idealized model underestimates the film thickness necessary for the recovery of AF ordering since it neglects the density fluctuations. One should note, that the temperature of the order–disorder transition in an open system should converge to the lower temperature, not higher than $T_{tr}(u_{AB}-1.0$. For example, the idealized model predicts that at T=0.36, the film with three filled layers is orientationally disordered, while any thicker film becomes orientationally ordered. On the other hand, the local density profiles $\rho_{k}\left(z\right)$, recorded along the adsorption isotherm recorded at T=0.36, have shown that the film with four occupied layers is still disordered and the ordering is restored in the film only after the formation of the fifth layer. The recovery of the ordering in thick films implies that complete wetting occurs since the bulk phase is also ordered at temperatures below the triple point. On the other hand, at T=0.37, the thermal excitations become high enough to hinder the recovery of the AF ordering in the film when the chemical potential approaches the bulk coexistence. Since the coexisting bulk condensed phase is still ordered, the disordered film cannot reach a large thickness and only incomplete wetting will occur. The results presented above confirm our claim that the dewetting transition below the triple point temperature results from low stability of the AF phase in the adsorbed films.

## 6. Summary and Final Remarks

In this paper, we have discussed the wetting scenarios in a simple lattice fluid with orientation-dependent interactions between the nearest neighbors and demonstrated that the main factors determining the possibility of complete wetting are the structures of the coexisting bulk and adsorbed phases and the strength of the surface potential.

When the surface potential is strong enough to ensure complete wetting at T=0, all systems undergo the dewetting transition. However, the mechanism of this transition depends on the bulk phase behavior. In the case of $u_{AB}=0$ and $u_{BB}\leq 0$, the bulk system does not exhibit the gas–liquid transition, but only the first-order transition between the dilute disordered and the orientationally ordered SAF phases over the temperatures between zero and $T_{o}\left(u_{BB}\right)$. In non-uniform systems, complete wetting occurs only when the growing adsorbed layer is ordered. When the surface potential becomes stronger, the adsorbed films disorder at the gradually increasing temperatures. As soon as the film becomes disordered, the dewetting transition takes place. The effect of the surface potential strength on the stability of ordered films and hence on the dewetting temperature, was confirmed by the results obtained for the adsorption at the selective surfaces. The temperature of the dewetting transition is independent of which side of the particle, A or B, is oriented toward the surface in the first layer, since both orientations lead to the same stability of the SAF structure.

**Figure 24:**
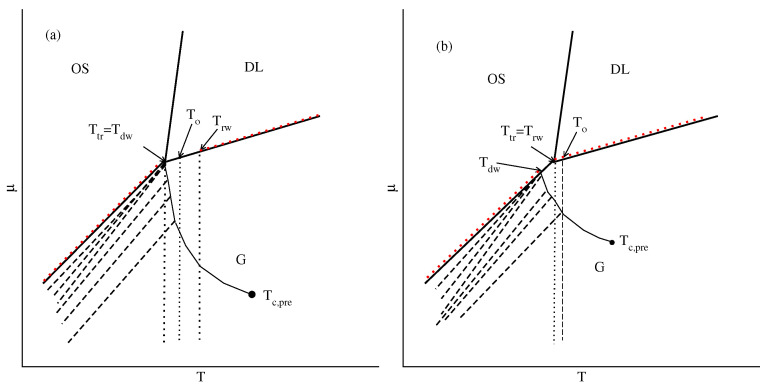
Schematic phase diagrams for the systems which exhibit the gas–liquid transition and order into the SAF (**a**) and AF (**b**) phase in the bulk. Thick solid lines mark the bulk phase boundaries between the gas (G), ordered solid (OS) and disordered liquid (DL) phases. The thick dashed lines mark layering transitions. The prewetting transition terminates at the critical point $T_{c,pre}$. Red dotted lines along the bulk phase boundaries mark the regions of complete wetting.

Other bulk systems ordering into the SAF phase at the sufficiently low temperatures, with $u_{AB}=u_{BB}$ and with $u_{BB}=0$ and $u_{AB}>-0.5$, exhibit the gas–liquid transition over a certain range of temperatures, between the triple and critical points. The presence of a liquid phase has a great influence on the wetting. The high stability of SAF ordering causes the adsorbed films to remain ordered even at temperatures above the bulk triple point. This gives rise to a triple point dewetting transition. However, the adsorbed phase loses the orientational order at the temperatures below the bulk critical point and this leads to the appearance of the reentrant first-order wetting transition. Figure 24 presents a schematic phase diagram for such systems. Our results agree with the theoretical predictions concerning the wetting behavior in the systems in which the solid and liquid adsorbed films wet the surface and the solid-like film remains stable at temperatures exceeding the bulk triple point [8]. To some extent, our findings are similar to the results of experimental study of the oxygen adsorbed on graphite [56,57]. Notwithstanding, the origin of the dewetting and the wetting transitions in the just-mentioned experimental system and the lattice gas model is quite different. In the adsorbed oxygen, it results from the presence of two different structures of solid oxygen (β and γ), while in the present model it is due to the formation of the orientationally ordered and disordered phases.

The wetting behavior changes when the bulk orders into the AF phase at low temperatures, up to the triple point. The stability of the AF ordering in the adsorbed films is lower than in the bulk. Therefore, the dewetting transition occurs at temperatures below the bulk triple point. However, complete wetting is restored at the triple point temperature since the bulk and adsorbed phases are liquid-like. Figure 24b shows the phase diagram expected for the systems with the surface potential strong enough to ensure complete wetting in the ground state. Here, we should recall the study of the Potts lattice gas model by Ebner [58], who obtained similar results qualitatively.

Figure 24 demonstrates that the only qualitatave difference between the systems ordering into SAF and AF phases is the location of dewetting transition, resulting from the changes in the stability of surface ordered phases.

When the surface potential is too weak for complete wetting to occur at T=0, the behavior of all considered systems is qualitatively the same and depends primarily on the ratio $\bar{u}/V_{o}$, with $\bar{u}$ being the energy of pair interaction, averaged over all orientations. This is so because the wetting transition occurs at rather high temperatures, at which the adsorbed films are orientationally disordered. Therefore, the observed wetting behavior is qualitatively the same as predicted for systems with isotropic interactions [3,9]. For a given value of $\bar{u}$, the wetting temperature gradually approaches the critical temperature when the surface potential becomes weaker. Of course, there is a threshold value of $V_{o}$, for which the wetting temperature meets the critical temperature.

Although the present model does not resemble any system accessible experimentally, it does bring some important results regarding the interplay between the wetting transitions and the stabilities of the ordered surface and bulk phases. In particular, we have shown that when the ordered structure in the adsorbed layer remains stable at the temperatures beyond the bulk triple point, the triple point dewetting occurs and complete wetting may be restored at higher temperatures. On the other hand, when the film disorders below the bulk triple point, the dewetting transition also occurs at the temperature below the triple point, and reentrant wetting occurs at the triple point. It is of interest to study the wetting of particles with the orientation-dependent interactions in the off-lattice systems. The density functional study of the amphiphilic Janus particles at the walls [59] has shown that the wall induces ordering in the adsorbed films and that this ordering depends on the strength of the surface potential felt by the hydrophobic and the hydrophilic parts of the Janus-like particles. The recent experimental and molecular dynamics study of Banik et al. [60] produced some interesting results on the substrate wettability guided orientational ordering of the Janus particles at surfaces. The results presented in this paper should be treated as the first step of the project aiming at a better understanding of the wetting transitions in the complex systems involving the orientation-dependent fluid–fluid and fluid–substrate interactions.

## Data Availability

Not applicable.
